# Dysregulated Lipid Metabolism and Its Role in α-Synucleinopathy in Parkinson’s Disease

**DOI:** 10.3389/fnins.2019.00328

**Published:** 2019-04-11

**Authors:** Irina Alecu, Steffany A. L. Bennett

**Affiliations:** ^1^Neural Regeneration Laboratory, Department of Biochemistry, Microbiology and Immunology, Ottawa Institute of Systems Biology, University of Ottawa, Ottawa, ON, Canada; ^2^Department of Cellular and Molecular Medicine, Brain and Mind Research Institute, University of Ottawa, Ottawa, ON, Canada; ^3^Department of Chemistry and Biomolecular Sciences, Centre for Catalysis and Research Innovation, University of Ottawa, Ottawa, ON, Canada

**Keywords:** Parkinson’s disease, α-synuclein, lipids, sphingolipids, glycerophospholipids, gangliosides, fatty acids, glucocerebrosidase (GBA)

## Abstract

Parkinson’s disease (PD) is the second most common neurodegenerative disease, the main pathological hallmark of which is the accumulation of α-synuclein (α-syn) and the formation of filamentous aggregates called Lewy bodies in the brainstem, limbic system, and cortical areas. Lipidomics is a newly emerging field which can provide fresh insights and new answers that will enhance our capacity for early diagnosis, tracking disease progression, predicting critical endpoints, and identifying risk in pre-symptomatic persons. In recent years, lipids have been implicated in many aspects of PD pathology. Biophysical and lipidomic studies have demonstrated that α-syn binds preferentially not only to specific lipid families but also to specific molecular species and that these lipid-protein complexes enhance its interaction with synaptic membranes, influence its oligomerization and aggregation, and interfere with the catalytic activity of cytoplasmic lipid enzymes and lysosomal lipases, thereby affecting lipid metabolism. The genetic link between aberrant lipid metabolism and PD is even more direct, with mutations in *GBA* and *SMPD1* enhancing PD risk in humans and loss of *GALC* function increasing α-syn aggregation and accumulation in experimental murine models. Moreover, a number of lipidomic studies have reported PD-specific lipid alterations in both patient brains and plasma, including alterations in the lipid composition of lipid rafts in the frontal cortex. A further aspect of lipid dysregulation promoting PD pathogenesis is oxidative stress and inflammation, with proinflammatory lipid mediators such as platelet activating factors (PAFs) playing key roles in arbitrating the progressive neurodegeneration seen in PD linked to α-syn intracellular trafficking. Lastly, there are a number of genetic risk factors of PD which are involved in normal lipid metabolism and function. Genes such as *PLA2G6* and *SCARB2*, which are involved in glycerophospholipid and sphingolipid metabolism either directly or indirectly are associated with risk of PD. This review seeks to describe these facets of metabolic lipid dysregulation as they relate to PD pathology and potential pathomechanisms involved in disease progression, while highlighting incongruous findings and gaps in knowledge that necessitate further research.

## Introduction

Parkinson’s disease (PD) is the second most common neurodegenerative disease and is projected to affect up to 9 million people worldwide by 2030 (Dorsey et al., 2007). While 5–10% of PD cases have a genetic basis, referred to as familial PD, 90–95% are defined as sporadic/idiopathic and have unknown etiology, involving a complex interplay of environmental factors and the genome. The familial form of PD has an earlier age of onset (<50 years), progresses faster, and is generally more severe than the sporadic form (Klein and Westenberger, 2012). Monogenic familial PD is caused by mutations in six genes: *SNCA, Parkin, LRRK2, DJ-1, PINK1, ATP13A2* (Klein and Westenberger, 2012), with some overlap between the genes involved in familial and sporadic PD respectively, mainly *SNCA* and *LRRK2* (van der Brug et al., 2015; Verstraeten et al., 2015). The greatest risk factor, however, for both PD and the related α-synucleinopathy Dementia with Lewy Bodies (DLB), is mutations in the glucosylceramidase-beta (*GBA*) gene, which encodes the lysosomal enzyme glucocerebrosidase (GCase) (Clark et al., 2010; Tsuang et al., 2012), implicating aberrant sphingolipid metabolism in PD pathogenesis. As *GBA* is also the genetic determinant of Gaucher Disease, differential disruption of sphingolipid metabolic pathways likely dictates disease penetrance and phenotype. Therefore, elucidating disease-specific metabolic disruptions provides a new avenue for cause-directed treatment of defining metabolic determinants.

The main pathological hallmark of PD is the accumulation of α-synuclein (α-syn), encoded by *SNCA*, and the formation of filamentous aggregates called Lewy bodies in the brainstem, limbic system, and cortical areas. This is accompanied by the progressive loss of dopaminergic neurons in the substantia nigra and the consequent reduction of dopamine in the striatum which leads to motor dysfunction. The classic motor symptoms of PD are resting tremor, rigidity, shuffling gait, and bradykinesia (slow movements). The symptoms are progressive, but the rate of deterioration in patients is variable (Fritsch et al., 2012). While PD is primarily a movement disorder, it is also associated with numerous non-motor symptoms which may arise much earlier in the course of the disease (Kalia et al., 2015). Non-motor symptoms include a combination of sensory and sleep disturbances, olfactory deficits, autonomic dysfunction, and neuropsychiatric symptoms such as visual hallucinations (Jain, 2011). According to the Braak staging scheme, the temporal mesocortex and neocortex, along with distinct areas of the brainstem, become progressively involved over time, with the substantia nigra (SN) being severely affected by stage 4 as well as some effects on the amygdala, while at stage 5 the anterior cingulate cortex (ACC) becomes affected (Braak et al., 2003).

The Lewy Body Dementias, DLB and PD with Dementia (PDD) are forms of dementia-parkinsonisms that overlap significantly with Alzheimer’s disease (AD) and PD. Both are defined by the deposition of AD-associated amyloid-β plaques and the intraneuronal accumulation of PD-associated Lewy bodies. PDD and DLB exhibit few differences on postmortem pathological examination and there are no laboratory-based biomarkers capable of discriminating these disorders (Chahine et al., 2014). Differential diagnosis rests solely on clinical observations (Henchcliffe et al., 2011). DLB is diagnosed when cognitive impairment, hallucinations, and aggressive dream enactments either precede or manifest within 12 months of the onset of parkinsonisms (Henchcliffe et al., 2011). PDD is diagnosed when patients exhibit these same signs of dementia at least 12 months after parkinsonisms (Henchcliffe et al., 2011). Mutations in *GBA* are associated with increased risk of PD and DLB (Clark et al., 2010; Tsuang et al., 2012). Heterozygous *GBA* mutations are found in ∼25% of all DLB and >10% of PD patients (Clark et al., 2010; Tsuang et al., 2012). Defining how these mutations are responsible for progressive critical metabolic impairments that precipitate PD, DLB, and PDD, represents a novel, potentially transformative, means of identifying persons at risk of imminent decline and developing new therapeutic avenues including substrate reduction and small molecule enzyme enhancement to alter their prognosis as reviewed below.

As with numerous other diseases, animal models have played an important role in elucidating various aspects of the pathomechanism of PD. The majority of these models use toxins that affect mitochondrial functions, such as 1-methyl,4-phenyl-1,2,3,6 tetrahydropyridine (MPTP) and 6-hydroxydopamine (6-OHDA). MPTP is actively taken up into nigrostriatal neurons, where it inhibits mitochondrial oxidative phosphorylation and causes cell death (Singer et al., 1987), while 6-OHDA accumulates in the cytosol of dopaminergic neurons and inhibits mitochondrial respiration (Glinka et al., 1997). We will be referring back to these two models throughout this review to point out how they have contributed to the knowledge of lipid changes in PD. Genetically modified models with disrupted genes known to be involved in PD are also used (Meredith et al., 2008), specifically relevant to this review, those incorporating lipid regulatory genes (Sardi et al., 2011, 2017; Tayebi et al., 2017).

Lipids are implicated in many aspects of PD pathology ranging from specific cytotoxic interactions with α-syn, to mutations in enzymes involved in lipid metabolism genes enhancing PD risk, lipid pathway alterations, and lipid involvement in oxidative stress and inflammation. This review will describe all of these aspects and point toward how the study of lipids in PD may provide novel answers both as potential biomarkers and novel treatment targets in the future.

## Lipid Interactions With α-Synuclein in the Pathogenesis of Parkinson’s Disease

α-syn is a small cytosolic protein that is highly expressed in the brain and is mainly located in synaptic terminals. It is composed of three domains: (1) a C-terminal region which is rich in aspartate and glutamate; (2) an internal hydrophobic domain; and (3) an amphipathic N-terminal domain (Golovko et al., 2009). There are several genetic variants for the α-syn locus related to both familial and sporadic PD, with A53T, A30P, and E46K being one of the most common mutations (Ostrerova-Golts et al., 2000; Markopoulou et al., 2008; Gispert et al., 2015). α-syn is present ubiquitously in all major brain cell types, including astrocytes (Cheng and Trombetta, 2004; Castagnet et al., 2005), microglia (Austin et al., 2006; Papadopoulos et al., 2006), and oligodendroglia (Richter-Landsberg et al., 2000; Mori et al., 2002). The physiological function of α-syn remains largely unknown (Jo et al., 2000). Based on its widespread distribution, it has been suggested that α-syn may play a wide number of roles in the nervous system, including regulating lipid metabolism (Jenco et al., 1998; Sharon et al., 2003a; Payton et al., 2004; Castagnet et al., 2005; Golovko et al., 2005, 2006, 2007; Narayanan et al., 2005; Barcelo-Coblijn et al., 2007), inflammatory response (Dalfo et al., 2004; Austin et al., 2006; Papadopoulos et al., 2006), the mobilization of synaptic vesicles (Murphy et al., 2000; Cabin et al., 2002; Dalfo et al., 2004), the control of neurotransmitter release (Abeliovich et al., 2000; Gureviciene et al., 2007; Nemani et al., 2010; Tsigelny et al., 2012; Wang et al., 2016), as well as modulating dopamine biosynthesis (Perez et al., 2002; Sidhu et al., 2004) and transport (Lee et al., 2001; Wersinger and Sidhu, 2003). A role in neuronal development and in synaptogenesis has also been proposed since α-syn appears early in murine brain development and is redistributed from the cytosol to the nerve terminals (Hsu et al., 1998). Recently it was demonstrated that α-syn also has a role in mitochondrial function, specifically as a physiological modulator of ATP synthesis by altering the efficiency of ATP synthase (Ludtmann et al., 2016).

**FIGURE 1:**
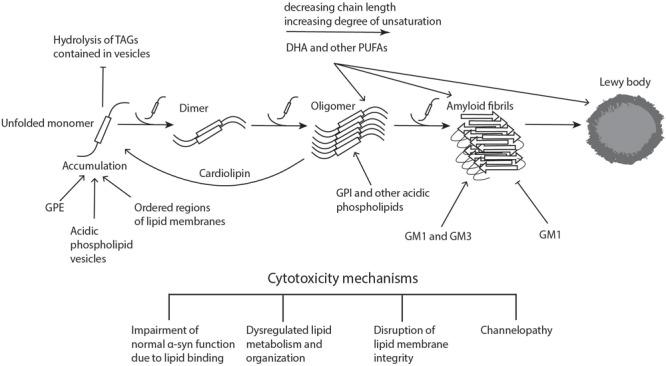
Lipid involvement in the aggregation and propagation of α-synuclein. Upon accumulation of unfolded α-syn, the monomers interact to form dimers, which can further grow to oligomers. These processes can take place both in the cytoplasm and in association with different cellular membranes. When soluble monomers continue to attach to oligomers, this eventually gives rise to amyloid fibrils, which can accumulate and form proteinaceous inclusions called Lewy bodies. α-Syn accumulation, oligomerization, and fibrillogenesis is highly affected by the lipid composition of the membranes it binds to, with a number of lipid species enhancing various steps of the process, as indicated on the diagram. The α-synuclein oligomers and fibrils formed are highly cytotoxic, leading to neurodegeneration. Cardiolipin is able to pull α-syn monomers from oligomeric fibrils, thereby buffering the toxicity. DHA, docosahexaenoic acid; GM1 and GM3, gangliosides; GPE, glycerophosphoethanolamine; GPI, glycerophosphoinositol; PUFAs, polyunsaturated fatty acids; TAGs, triacylglycerols. Figure was adapted from Lashuel et al. (2013).

An extensive body of research has accumulated over the past 20 years concerning α-syn membrane binding. It has been well established that α-syn is disordered in solution, but it can assume an α-helical conformation upon lipid membrane binding (Davidson et al., 1998; Zhu and Fink, 2003). As early as 1988, the association of α-syn with lipids became apparent when it was found to co-localize with synaptic vesicles, a finding that has been validated by multiple laboratories (Maroteaux et al., 1988; Sharon et al., 2003a; De Franceschi et al., 2011). The binding of α-syn to lipids has been well characterized in a number of lipid systems (see Figure 1 for summary of α-Syn lipid interactions leading to aggregation) (Eliezer et al., 2001; Bodner et al., 2009; Fusco et al., 2014). Moreover, lipidomic assessments have shown that specific lipid-α-syn complexes are required to enhance α-syn binding to synaptic membranes, highlighting 1-O-hexadecyl-2-acetyl-sn-glycero-3-phosphocholine [PC(O-16:0/2:0)]^1^, a platelet activating factor (PAF) glycerophosphocholine, as one of these principal lipid second messenger components (Wislet-Gendebien et al., 2008). However, less information exists on the modulation of the kinetics of conversion of monomeric α-syn into amyloid fibrils by different membrane lipid compositions (Zhu et al., 2003; Fink, 2006; Martinez et al., 2007). Moreover, most of these studies have been performed either in the presence of catalyzing polymer surfaces (Grey et al., 2011) and/or under mechanical agitation (Zhu et al., 2003). These experiments therefore do not fully elucidate the endogenous mechanism of α-syn aggregation, as in these conditions the protein also aggregates in the absence of lipids. Galvagnion et al. (2016) used protein-repellant surfaces to undertake a systematic study of α-syn binding to different model membranes (Galvagnion et al., 2016). Their results indicated that the efficiency of α-syn binding was dependent on the membrane fluidity. α-Syn showed an affinity for negatively charged phospholipids, in addition to complexing with small bioactive glycerophosphocholine second messengers including the PAF, PC(O-16:0/2:0) with aggregation enhanced in the presence of lipid species with short hydrocarbon chains (Wislet-Gendebien et al., 2008; Galvagnion et al., 2016). This observation may have pathological implications, as lipids with short hydrocarbon chains can be formed during polyunsaturated lipid peroxidation, a process which is very damaging to cells and which affects membrane fluidity (Beranova et al., 2010; Negre-Salvayre et al., 2010).

### Physiological Aspects of α-Synuclein Interactions With Lipids

Binding of α-syn to lipids appears to be necessary for many of α-syn’s proposed physiological roles. Upon binding to lipid vesicles, α-syn undergoes a major structural transition from random coil to α-helical structure (Davidson et al., 1998), supporting the possible role of α-syn in lipid binding and transport. Cole et al. (2002) reported that α-syn accumulated on phospholipid monolayers surrounding lipid droplets that contained high levels of triacylglycerols (TAGs). Accumulation was seen in both HeLa cells that had been treated with high fatty acid concentrations as well as primary hippocampal neurons. The accumulated α-syn prevented the stored TAGs from being hydrolyzed. In contrast, when the same experiment was performed in cells with PD mutant α-syn, there was variable distribution on the lipid droplets which resulted in higher levels of TAGs being hydrolyzed (Cole et al., 2002). The PD mutant α-syn A30P showed no lipid droplet binding, while A53T did bind but was not able to protect the stored TAGs from hydrolysis (Cole et al., 2002). These results suggest that wild-type α-syn is able to protect lipid droplets from neutral lipases or directly inhibit them, while mutant α-syn loses this ability. Although the A53T mutation does not impair the binding of the α-syn to lipid droplets, it could alter the conformation of the protein in a way that renders it ineffective in preventing TAG hydrolysis. Jo et al. reported that wild-type α-syn bound to acidic phospholipid vesicles and this binding was significantly augmented by the presence of glycerophosphoethanolamine (GPE), a family of neutral phospholipids (Jo et al., 2000). The association of soluble wild-type α-syn with planar lipid bilayers resulted in the formation of aggregates and small fibrils. The PD mutant α-syn A53T induced similar disruption in the lipid membranes, although at a slower rate.

α-Syn lipid binding also appears to play a role in mitochondrial function. As will be discussed in more detail in Section “Binding of α-syn With Mitochondrial Membranes,” wild-type α-syn has been demonstrated to bind to cardiolipin, a specific phospholipid in the inner mitochondrial membrane (Guardia-Laguarta et al., 2014; Robotta et al., 2014). By creating a triple synuclein knock-out to avoid confounding effects of β-synuclein and γ-synuclein, Ludtmann and colleagues demonstrated that α-syn plays a role in modulating mitochondrial bioenergetics by interacting with ATP synthase and increasing its efficiency (Ludtmann et al., 2016). Therefore, it appears that the binding of α-syn to cardiolipin in the mitochondrial membrane its part of its physiological role as related to mitochondrial metabolism.

A number of studies have shown α-syn to also have a function in lipid metabolism. It has structural similarities to class A2 lipoproteins (George et al., 1995; Davidson et al., 1998), some sequence similarity to fatty acid binding protein (FABP) (Sharon et al., 2001), and is present in considerable amounts in microsomes, where complex lipid metabolism occurs (Sharon et al., 2001; Golovko et al., 2006). The lipid-binding domain of α-syn, which adopts a secondary structure very similar to that of phospholipase A2 proteins, in fact mediates multimerization induced by polyunsaturated fatty acids (PUFAs) (Perrin et al., 2001). The specific effects that α-syn has on different aspects of lipid metabolism will be discussed in detail in Section “The Role of α-syn in Lipid Metabolism.”

Taken together, these results suggest that the interaction of α-syn with lipids is physiologically important, and that PD-associated mutations may impair the normal function of the protein.

### Pathological Aspects of α-Synuclein Lipid Binding

Lipid binding has also been shown to play an integral role in the pathological aspects of α-syn, namely by augmenting α-syn multimerization. α-Syn multimerization and subsequent fibril growth are believed to play central roles in PD pathogenesis (Conway et al., 1998; Taschenberger et al., 2012). Initially Davidson and colleagues showed that α-syn preferentially bound to vesicles containing acidic phospholipids, but not to those with neutral phospholipids (Davidson et al., 1998). Stable multimers formed upon exposure to glycerophosphoinositols, the most acidic phospholipids (Davidson et al., 1998). Interestingly, one of the important functions of glycerophosphoinositols is to modulate vesicle cycling at presynaptic terminals where α-syn is enriched (Frere et al., 2012). Further studies determined that α-syn oligomerization was also enhanced in the presence of PUFAs (Perrin et al., 2001). In mesencephalic neuronal cells treated with fatty acids, increasing the degree of unsaturation of the fatty acids dramatically increased the amount of soluble α-syn oligomers, while treatment with fully saturated fatty acids lowered levels of oligomeric α-syn (Sharon et al., 2003b). The length of the carbon chain also affected the amount of oligomerization, although to a smaller extent than did degree of saturation. In the same study, Sharon et al. showed that the soluble α-syn oligomers then associated into insoluble high-molecular weight complexes. Assayag et al. (2007) further expanded on these findings, demonstrating that exposure of dopaminergic neuronal cell lines overexpressing α-syn to PUFAs resulted in the formation of α-syn oligomers, and later led to the development of Lewy-like proteinaceous inclusions in the cytoplasm. Furthermore, they reported that the α-syn oligomers were associated with cytotoxicity, although the existence of the Lewy-like aggregates seemed to be protective. Therefore, the types of lipids in the cellular environment play a critical role in the aggregation of α-syn suggesting that dysregulation of lipid levels and lipid pathways may be an important contributing factor in the pathogenesis of PD.

Polyunsaturated fatty acids are essential in maintaining neuronal membrane fluidity and permeability (Ruiperez et al., 2010). They also play a number of critical roles within the cell, including the activation of phospholipases (Kim et al., 1999), recycling of synaptic vesicles (Schmidt et al., 1999), and the inhibition of ion channels (Leaf et al., 1999). Arachidonic acid (ARA) and docosahexaenoic acid (DHA) are the most enriched PUFAs in the human brain (Chen et al., 2008). α-Syn has been reported to immediately change its structure in the presence of both of ARA and DHA, which are released upon glycerophospholipid hydrolysis, taking on its α-helical conformation (Broersen et al., 2006; De Franceschi et al., 2009). Upon longer exposure to DHA, α-syn gradually assembles into amyloid-like fibrils, with the DHA itself being part of the aggregate (Broersen et al., 2006). DHA accounts for 60% of glycerophospholipid esterified fatty acids in the plasma membrane, making it an important factor to consider in the potential aggregation of α-syn and its potential functions (Lukiw and Bazan, 2008). Furthermore, the level of α-syn gene expression is increased upon elevated DHA intake, and the consequently formed oligomers are toxic to cells (De Franceschi et al., 2009, 2011).

Another family of lipids, the gangliosides, have also been shown to accelerate the kinetics of α-syn conversion to amyloid fibrils (Grey et al., 2015), specifically the gangliosides GM1 and GM3. Gangliosides are composed of monosaccharide groups attached to a ceramide backbone and serve as precursors to the complex gangliosides which are abundant in the brain (Proia, 2004). The interaction of gangliosides with amyloid-β, the protein associated with AD pathology, has been well established (Yanagisawa et al., 1995; Hayashi et al., 2004; Hoshino et al., 2013), with elevated ganglioside levels being present in the brain and cerebrospinal fluid of AD patients. Similar results were reported by Grey et al. (2015) for α-syn, showing that both GM3 and GM1 accelerated its aggregation, while the rest of the glycerophospholipids they tested slowed down the aggregation. However, a study by Martinez et al. (2007) somewhat contradicted these findings, showing that in fact GM1-containing small unilamellar vesicles inhibited the formation of α-syn fibrils by inducing and maintaining its α-helical conformation for both wild-type and A53T mutant α-syn. Vesicles containing GM2 or GM3 gangliosides showed much weaker inhibitory effects. These findings are in line with numerous studies showing that treatment with GM1 improves both cognitive and motor deficits in PD animal models and in PD patients (Hadjiconstantinou et al., 1986, 1989; Schneider and Distefano, 1995; Schneider et al., 1995, 2010, 2013). These findings will be discussed more in detail below in “Lipids as Targets for PD Treatment.”

### Potential Mechanisms Leading to Cytotoxicity Upon α-Synuclein Lipid Binding

The observation that the concentrations of certain fatty acids and more complex lipids can enhance α-syn aggregation and toxicity suggest alternative therapeutic strategies that could be undertaken to treat PD. Understanding why and how these lipids augment cytotoxicity will allow for a deeper understanding of the pathomechanism at play and will allow for more specific and personalized treatment targets. One potential mechanism of cytotoxicity is the disruption of lipid membranes. Fecchio et al. (2013) showed that the toxicity of α-syn and DHA oligomers to cells is due to the disruption of the integrity of lipid membranes. Through their binding to negatively charged vesicle membranes, the oligomers cause leakage of small molecules out of the vesicles (MW 0.6 kDa). In dopaminergic SH-SY5Y cells, treatment with α-syn and DHA oligomers increased the permeability to propidium iodide (Fecchio et al., 2013). In oligodendroglial cells overexpressing the α-syn mutation A53T, supplementation with DHA followed by oxidative stress due to hydrogen peroxide led to the formations of α-syn fibrils and a decrease in α-syn solubility. Oligodendroglial cells expressing wild-type α-syn displayed the same changes, therefore the effect is largely attributable to the presence of DHA and/or oxidative stress (Riedel et al., 2011). These results indicate that lipid membrane disruption may be one of the mechanisms of toxicity of α-syn oligomers leading to PD. Lipid membrane integrity is particularly important in neuronal cells, as action potential firing is mediated by changes in membrane potential regulated by voltage-gated channels, as well as the maintenance of sodium and potassium gradients.

Another potential mechanism of induced cellular toxicity due to the interaction of PUFAs with α-syn could be the incorporation of the PUFAs themselves within the complexes, as has been shown for DHA, which would sequester and compartmentalize PUFAs thereby affecting their normal roles within the cell (Broersen et al., 2006). Supporting this hypothesis, Jenco et al. (1998) showed that α-syn inhibits the activation of phospholipase D2 via PIP_2_, which hydrolyses glycerophosphocholines to produce phosphatidic acid and choline. Therefore, the incorporation of PUFAs within α-syn aggregates could lead to toxicity by affecting PUFA metabolism and organization within the cell membrane.

Binding of α-syn with lipids may also affect the normal lipid function. A ganglioside-binding domain (GBD), which has a marked preference for GM3, has been identified in α-syn (Di Pasquale et al., 2010). One of the residues in this domain is mutated in a familial form of PD (E46K). This domain is structurally related to the glycosphingolipid-binding domain shared by a variety of amyloid protein, including β-amyloid peptide which is involved in the pathology of AD (Di Pasquale et al., 2010). While the domain identified in α-syn was found to interact with a number of glycosphingolipids, it had a distinct preference for the ganglioside GM3. The α-syn mutant E46K was demonstrated to have a stronger affinity for GM3 than the wild-type protein. When the E46K protein was incubated with reconstituted glycerophosphocholine bilayers, the channels formed were functionally impaired compared to those formed by wild-type α-syn. When GM3 was present in the reconstituted bilayers, this channelopathy was no longer observed (Di Pasquale et al., 2010).

Taken together, it is clearly important to elucidate the physiological role of α-syn in the normal brain as well as the pathological role of mutant α-syn in PD in order to be able to develop treatments that can impede the pathological aspects while not affecting the physiological roles that are necessary for neuronal function.

## Binding of α-syn With Mitochondrial Membranes

Mitochondrial dysfunction has been shown to be involved in PD pathogenesis, but the underlying mechanisms involved have not yet been elucidated. Both genetic predisposition to PD and environmental risk factors play roles in different aspects of mitochondrial function, including bioenergetic capacity, dynamic morphological changes during fission and fusion, and transport (Kamp et al., 2010; Burte et al., 2015; Ryan et al., 2015). The lipid environment within mitochondrial membranes influence all of these processes as they determine membrane curvature and structure, as well as affecting the recruitment and activity of specific proteins (Aufschnaiter et al., 2017). The role of dysfunctional mitochondria in the pathogenesis of PD has been demonstrated in several models, including aging yeast (Buttner et al., 2008), *Drosophila* (Greene et al., 2003), as well as in Parkin-deficient transgenic mouse models (Palacino et al., 2004; Shim et al., 2011).

As previously mentioned, α-syn can tightly interact with various artificial membranes, but it associates much more weakly with native membranes (George et al., 1995; Kahle et al., 2000). Nakamura et al. (2008) reported that α-syn preferentially binds to mitochondrial membranes *in vivo* while Guardia-Laguarta et al. (2014) showed that in cell lines and in brain tissue from mice and humans, wild-type α-syn was present specifically in mitochondria-associated endoplasmic-reticulum membranes (MAMs). The interaction between α-syn and these membranes appears to initially require an anionic charge (Cole et al., 2008). Cardiolipin, a mitochondrial-specific lipid, has a diphosphatidyl glycerol headgroup which imparts an anionic charge to it. By using artificial membranes with and without cardiolipin, Nakamura et al. (2011) showed that this acidic phospholipid is essential for interactions with α-syn. Quenching of the anionic headgroup inhibits the association of α-syn with artificial mitochondrial membranes (Cole et al., 2008). The acyl side chains on cardiolipin induce negative curvature in mitochondrial membranes and, along with the anionic headgroup, have been reported to facilitate α-syn docking onto the membrane by physically interacting with the N-terminal region of wild-type α-syn (Grey et al., 2011; Zigoneanu et al., 2012; Ghio et al., 2016). Interestingly, this interaction is bi-directional, as the absence of α-syn dramatically reduces the concentration of both cardiolipin and its precursor phosphatidylglycerol (Ellis et al., 2005; Barcelo-Coblijn et al., 2007). Furthermore, there is an increase in saturated fatty acids bound to the glycerol backbone of cardiolipin in the absence of α-syn (Ellis et al., 2005).

As mentioned in Section “Pathological Aspects of α-synuclein Lipid Binding,” acidic phospholipids have been reported to facilitate α-syn aggregation (Davidson et al., 1998; Jo et al., 2000). Contrary to this, Ryan et al. (2018) recently showed that in human dopaminergic neurons, cardiolipin translocates to the outer mitochondrial and here binds to both A53T and E46K mutant α-syn, inducing it to take the α-helical conformation. Furthermore, the cardiolipin in the outer mitochondrial membrane pulled α-syn monomers from oligomeric fibrils and enabled their refolding back into α-helices, thereby buffering their aggregation and resultant pathological impacts (Ryan et al., 2018). Interestingly, in fibrils composed of mutant α-syn, this buffering capacity was reduced (Ryan et al., 2018). While these results are inconsistent with reports from early studies with regards to the interaction of acidic lipids with α-syn, it is likely that the effects observed may be specific to cardiolipin which is only found in mitochondria and MAM. All previous studies had been performed on membranes containing acidic phospholipids such as glycerophosphoinositols and glycerophosphoethanolamines (Davidson et al., 1998; Jo et al., 2000).

Aside from cardiolipin, GPE in mitochondria have also been recently linked to α-syn toxicity. Knockout of the enzyme which synthesizes GPE, phosphatidylserine decarboxylase Psd1 (mammalian Pisd), in yeast and worm models of PD was shown to result in the formation of α-syn foci, ER stress, defects in trafficking, and decreased respiration (Wang et al., 2014). The addition of ethanolamine, which is converted to GPE by the Kennedy pathway, was found to partially rescue these deleterious effects – α-syn foci were decreased and ER stress was reduced, but there was no influence on respiration (Wang et al., 2014).

To conclude, there is a close and bi-directional relationship between cardiolipin in mitochondrial membranes and α-syn, with changes in each one affecting the form or function of the other and the resulting pathological interactions. Other mitochondrial phospholipids, such as GPE, also appear to have an effect on α-syn toxicity. Therefore, mitochondrial lipids present a novel opportunity for the development of biomarkers and new therapeutic strategies in PD.

## The Interaction of α-syn With Lipid Rafts

Lipid rafts are microdomains in the plasma membrane that are liquid-ordered and enriched in sphingomyelin, cholesterol, and saturated fatty acid (SFAs), while having a low content of PUFAs. They promote lipid-lipid and lipid-protein interactions due to their highly saturated and liquid-ordered physicochemical properties (Brown and London, 2000; Pike, 2003; Pike, 2009), therefore playing a central role in intercellular communication and signal transduction. Alterations in lipid raft composition have been associated with abnormal neuronal function and neurotransmitter signaling (Allen et al., 2007; Marin et al., 2007; Ferrer, 2009; Ramirez et al., 2009).

A recent study monitored the conformational changes of α-syn upon binding to gel and liquid-ordered phases, specifically in the presence of glycerophosphocholine (O’Leary et al., 2018). The authors observed that the α-helical conformation of the protein was lost as the lipid phase transitioned from gel to fluid in zwitterionic membranes, while *N*-acetylation increased α-helicity in the presence of their immediate metabolites and precursors the lysophosphocholines, which lack a long-chain fatty acyl group at the *sn*-1 or *sn-*2 position. *N*-acetylation is a common post-translational modification of proteins in eukaryotes, and *N*-acetyl α-syn was reported to be the predominant form in brains of both healthy controls and PD patients (Anderson et al., 2006). There have been conflicting reports regarding the impact of N-acetylation on α-syn lipid binding. NMR studies showed that *N*-acetyl α-syn bound more strongly to the membrane (Maltsev et al., 2012; Dikiy and Eliezer, 2014), while one study reported no effect (Fauvet et al., 2012). Galvagnion and colleagues showed that α-syn aggregation in the presence of anionic phospholipids is also affected by membrane fluidity, with different aggregation propensities for saturated versus unsaturated lipid membranes (Galvagnion, 2017). These results suggest that ordered regions in biological membranes, such as lipid rafts, serve as locations for α-syn aggregation. Further supporting this, the localization of α-syn to synaptic terminals appears to be mediated by the presence of lipid rafts (Fortin et al., 2004) and complexing with small glycerophosphocholine seconds messengers (Wislet-Gendebien et al., 2008). More specifically, Fortin and colleagues found that the PD-associated α-syn mutation A30P blocked α-syn interaction with lipid rafts, suggesting a role of lipid rafts in the normal localization and consequent function of α-syn.

Further supporting the potential role of lipid rafts and their interaction with α-syn in the pathogenesis of PD, Fabelo et al. (2011) reported that lipid rafts isolated from the frontal cortices of patients with PD displayed profound lipid alterations compared to healthy controls. Plasmalogen, sulfatide, and cerebroside levels were greatly decreased in PD, whereas glycerophosphoserine and glycerophosphoinositol were higher (Fabelo et al., 2011). The deficiency of sulfatides and cerebrosides has been previously linked to the accumulation of ceramide, as this is formed by hydrolysis of sulfatides and cerebrosides. Increased ceramide levels have many deleterious effects, including disruption of the mitochondrial respiratory chain, upregulation of cytokines, and apoptosis (Hannun and Luberto, 2000). The depletion of sulfatides and consequent increase in ceramide levels has also been associated with the neurodegeneration seen in AD using unbiased lipidomic approaches (Cheng et al., 2003). However, it has been hypothesized that in PD, decreased ceramide levels are potentially pathogenic and have been reported to be decreased in post-mortem brain tissue of PD patients (Bras et al., 2008; Abbott et al., 2014). One potential explanation is that while levels of ceramide may be decreasing overall, there is a mechanism that attempts to maintain their levels in lipid rafts specifically. This hypothesis clearly requires subcellular fractionation and lipidomic assessment to monitor site-specific lipid metabolism.

Perturbations in the association of α-syn with lipid rafts may have a role to play in PD pathogenesis. More generally, fluid lipid membranes (liquid disordered and liquid crystalline) appear to play a role in preventing α-syn aggregation and maintaining its homeostasis, while perturbations in the lipid balance could be a PD risk factor.

## The Role of α-syn in Lipid Metabolism

**FIGURE 2:**
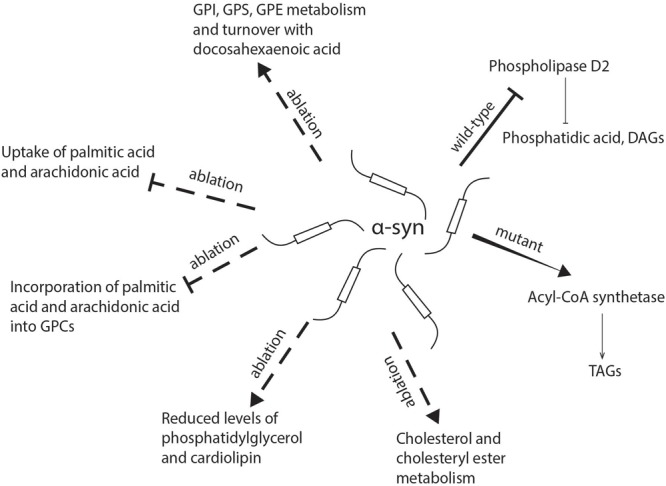
Schematic representation of the role of α-synuclein in lipid uptake and metabolism. α-syn deficiency inhibits the uptake of palmitic acid and arachidonic acid and their further metabolism into glycerophosphocholine, while there is an increase in the incorporation of docosahexaenoic acid into glycerophosphoethanolamine, glycerophosphoinositol, and glycerophosphoserine. The absence of α-syn also reduces levels of phosphatidylglycerol and cardiolipin in mitochondria. Mutant α-synuclein has been shown to enhance the activity of acyl-CoA synthetase and lead to an increased generation of triacylglycerols, while wild-type α-synuclein may inhibit phospholipase D2, which hydrolyzes glycerophospholipids into diacylglycerols and phosphatidic acid. DAG, diacylglycerol; FA, fatty acid; GPC, glycerophosphocholine; GPE, glycerophosphoethanolamine; GPI, glycerophosphoinositol; GPS, glycerophosphoserine; TAG, triacylglycerol.

α-syn has been shown to play a role in a number of different lipid metabolic pathways, with significant consequent implications for its toxicity. Willingham et al. (2003) conducted a genome-wine screening study in yeast which supports the involvement of lipid metabolism in α-syn toxicity. The authors found that 18 out of 57 genes which modified the toxicity of α-syn were related to lipid metabolism and vesicle-mediated transport. Here we will describe the lipid pathways that α-syn has been implicated in thus far (see Figure 2 for summary of the role α-syn in lipid uptake and metabolism).

### α-Synuclein Involvement in Fatty Acid Uptake and Metabolism

There is accumulating evidence that α-syn plays an important role in fatty acid uptake and metabolism which is specific for different fatty acids. In astrocytes isolated from α-syn gene-ablated mice, the uptake of [1-^14^C] labeled palmitic acid (16:0) and [1-^14^C] labeled ARA [20:4(n-6)] were significantly decreased (Golovko et al., 2005, 2006). α-Syn deficiency decreased the rate of incorporation of both palmitic acid and ARA into a number of different glycerophospholipid classes, an observation that was brain specific as no changes were seen in the liver (Golovko et al., 2005, 2006). A similar experiment was performed with labeled DHA which demonstrated that although α-syn deficiency did not affect the uptake of this fatty acid, there was an increase in the rate of incorporation and turnover of glycerophosphoinositol, glycerophosphoserine, and glycerophosphoethanolamine pools (Golovko et al., 2007). This effect is opposite that reported for palmitic acid and ARA and therefore suggests a compensatory mechanism in maintaining critical levels of glycerophospholipids in cells.

### α-syn Involvement in Triacylglycerol and Cholesterol Metabolism

Mutant α-syn was also recently implicated in the metabolism of TAGs. It was observed that the overexpression of A53T α-syn led to an increase in the levels of TAGs which was accompanied by enhanced activity of acyl-CoA synthetase, which catalyzes the formation of fatty acyl-CoA that serve as substrates in β-oxidation and glycerophospholipid and sphingolipid biosynthesis (Sanchez Campos et al., 2018). This is an interesting metabolic switch that could be used to monitor the onset of α-syn aggregation and consequent neurodegeneration.

α-syn deficiency has also been shown to impact cholesterol metabolism in brain and astrocytes, with cholesteryl esters and cholesterol being significantly elevated (Castagnet et al., 2005; Barcelo-Coblijn et al., 2007). This is an important finding with regard to PD pathology, as mature neurons depend on cholesterol synthesized and exported from astrocytes (Poirier et al., 1993; Dietschy and Turley, 2004), and when this is lacking neurons cannot form synapses in culture (Pfrieger, 2003) and presynaptic transmitter release is significantly decreased (Nagler et al., 2001).

### α-syn Effect on Phospholipase Activity

A number of reports confirm that α-syn interacts with and affects the activity of phospholipase D (PLD), but there is some divergence in the findings. PLD forms phosphatidic acid (PA) and diacylglycerol (DAG) by catalyzing the hydrolysis of head groups from glycerophospholipids (Tsai et al., 1989; Bocckino et al., 1991; Bauldry et al., 1992; Zhao et al., 1993; Ktistakis et al., 1995). Two isoforms of PLD, PLD1 and PLD2, have been characterized in most cell types and tissues. PLD1 has low basal activity (Hammond et al., 1995; Hammond et al., 1997), while PLD2 is more active and is insensitive to further stimulation by known PLD1 activators (Colley et al., 1997). It was hypothesized that PLD2 activity was attenuated by an unknown inhibitory factor. Jenco et al. (1998) attempted to describe this factor by purifying a protein that selectively inhibited PLD2 and through sequencing and immunological analysis found that this protein was a mixture of α- and β-synucleins. Later on, Gorbatyuk et al. (2010) showed that overexpressing PLD2 (gain of function) leads to loss of dopaminergic neurons in the striatum and neurodegeneration of the substantia nigra, and that α-syn co-expression suppressed PLD2 toxicity. However, *in vitro* α-syn showed no inhibitory function on the activity of PLD. Using a variety of systems and approaches, including purified proteins, PLD transfection in a number of mammalian cell lines, as well as a yeast system, Rappley et al. (2009) reported no significant inhibition of PLD by α-syn. Conversely, it has been reported that reducing PLD1 expression or inhibiting its enzymatic activity (loss of function) compromised the clearance of α-syn aggregates (Bae et al., 2014). Furthermore, the authors showed decreased PLD1 expression (loss of function) in the brains of patients with Lewy body dementia. Conde et al. (2018) recently showed that the overexpression of wild-type α-syn in human neuroblastoma cells inhibited the expression of PLD1 and affected ERK1/2 signaling, which appeared to alter the actin cytoskeleton and reduce the neurofilament light chain. These results raise the possibility that the modulation of both PLD1 and PLD2 activities is involved in the pathomechanism of PD and suggests that the level and modification of α-syn may affect these activities by controlling the cleavage of membrane lipids and membrane biogenesis.

Lipid homeostasis is fundamental in maintaining normal cellular functions. α-Syn appears to play a role in modulating many aspects of lipid metabolism ranging from fatty acid uptake to inhibition of enzyme activity. Therefore, the dysregulation of brain lipid metabolism by α-syn may play an important role in propagating PD pathology.

## Lipid Pathway Alterations in Parkinson’s Disease

**FIGURE 3:**
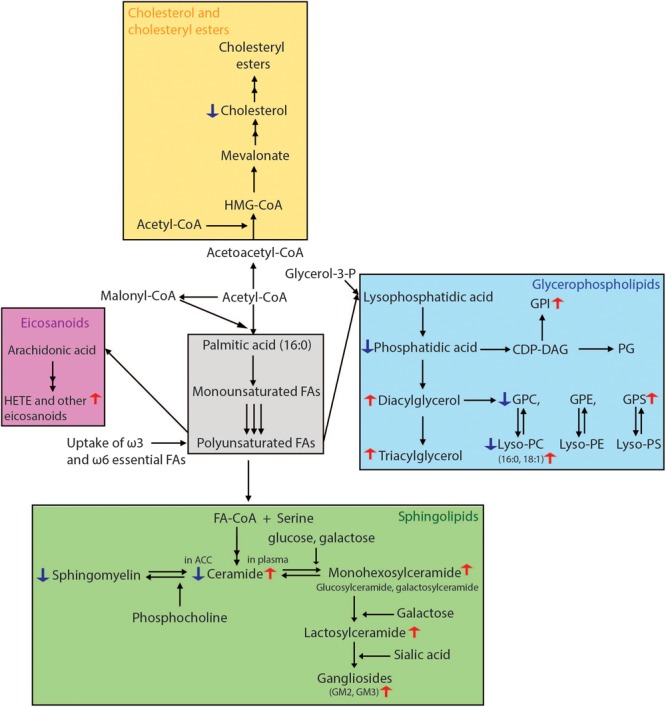
Overview of lipid biosynthetic and metabolic pathways indicating lipid changes which have been observed in Parkinson’s disease. Fatty acid biosynthesis begins with the conversion of acetyl-CoA to malonyl-CoA. The repeated condensation of these two fatty acyl-CoA’s results in palmitic acid, which is 16 carbons long and fully saturated. Monounsaturated fatty acids are then formed by the introduction of a double bond at carbon 9. PUFAs are generated by further desaturations and elongations. Glycerophospholipids result from the condensation of both saturated and unsaturated fatty acids with glycerol-3-phosphate. For the formation of sphingolipids, fatty acids require activation to acyl-CoA’s which then undergo condensation with serine. The attachment of various head groups like phosphocholine and hexosyl moieties gives rise to sphingomyelin and hexosyl-ceramides, respectively. Gangliosides are formed by the addition of a sialic acid to lactosyl-ceramides for GM3, as well as *N*-acetylgalactosamine to generate GM1 and GM2. Arachidonic acid, one of the two most enriched polyunsaturated fatty acids in the human brain, is used in the synthesis of HETE and other eicosanoids. For cholesterol synthesis, acetyl-CoA is first converted to acetoacetyl-CoA, followed by addition of another acyl group to form HMG-CoA. This is then processed into mevalonate, which through a number of subsequent reactions becomes cholesterol. Lipidomics methods employing liquid chromatography mass spectrometry have been able to reveal many lipid alterations in cells, post-mortem brain tissue and plasma from patients with PD which in the future could be developed for use as prognostic and diagnostic biomarkers. CDP-DAG, cytidine diphosphate-diacylglycerol; FA, fatty acid; HETE, hydroxyeicosatetraenoic acid; HMG-CoA, 3-methylglutaryl-3-hydroxy-CoA; GPC, glycerophosphocholine; GPE, glycerophosphoethanolamine; PG, phosphoglycerol; GPI, glycerophosphoinositol; GPS, glycerophosphoserine.

Understanding changes in lipid levels in brain areas both involved in PD and those spared, along with those in the periphery could elucidate novel pathways involved in PD pathogenesis as well as identify potential biomarkers for diagnosis and therapeutic monitoring (see Figure 3 for summary of lipid pathway alterations in PD).

### Lipid Changes in Animal Models of PD

Animal models of disease are one of the most important tools available for elucidating the cellular and molecular changes that happen and should reflect the changes observed in humans. Generally, information regarding lipid changes in animal models of PD is minimal. Farmer and colleagues used high performance liquid chromatography coupled with mass spectrometry to profile the sphingolipids and glycerophosphocholines in the substantia nigra of a 6-OHDA-induced mouse model of PD (Farmer et al., 2015). They found that 17 glycerophosphocholine and lysophosphocholine (LPC) species were significantly reduced in these mice. Interestingly, LPC(16:0/0:0) and LPC(18:1/0:0) were increased in the 6-OHDA-treated mice. These two LPC species were also found to be increased in human fibroblasts deficient in *Parkin* (Lobasso et al., 2017). As mentioned, mutations in the *Parkin* gene are well-known causes of PD and induce defects in mitochondria and dysfunctional autophagy. Lobasso et al. (2017) examined how *Parkin* mutations in primary human skin fibroblasts affected the lipidome of these cells. They reported that the *Parkin*-mutant fibroblasts had higher levels of glycerophosphoserine, glycerophosphoinositol, gangliosides GM2 and GM3, as well as LPC(18:1/0:0) and LPC(16:0/0:0) (Lobasso et al., 2017). It has been shown that these two species play a role in inflammatory signaling, and inflammation has been shown to be involved in PD pathogenesis (Cunningham et al., 2008), while the higher levels of glycerophosphoinositol and glycerophosphoserine may cause defects in mitochondrial turnover (Lobasso et al., 2017).

### Lipidomic Analysis of Pathological Lipid Changes in PD Patient Brains

Lipidomic analysis has the capability of offering a large-scale picture of lipid changes and elucidating network-wide effects. Using liquid-chromatography mass spectrometry, Wood et al. found that DAGs, with both monounsaturated and polyunsaturated hydrocarbon chains, are increased in the frontal cortex of PD patients (Wood et al., 2018). The greatest elevations were observed in the cohort with the most severe cortical neuropathology (subjects with moderate to frequent neocortical neuritic plaques). Wood et al. also reported a significant decrease in the levels of phosphatidic acid 16:0 in all three PD subgroups. DAGs are essential for the synthesis of glycerophospholipids and as second messengers in the nuclear lipid signaling pathway. DAG levels are tightly controlled by DAG kinase which converts DAGs to phosphatidic acids (Evangelisti et al., 2006). Therefore, the elevated DAGs and lower levels of lysophosphatidic acid suggest that there may be a dysfunction in the DAG kinase regulation of DAG steady-state levels in proteinopathies (Wood et al., 2018).

Using a case-control approach to analyze the comprehensive sphingolipidome, Abbott and colleagues analyzed the postmortem brain tissue from the ACC and from the occipital cortex (Abbott et al., 2014). While the ACC showed Lewy body pathology starting at Braak stage IV, the occipital cortex did not show any PD-related pathological changes. In the ACC of PD patients, total ceramide and sphingomyelin levels were approximately half of those in controls. These changes were not seen in the occipital cortex. Furthermore, a significant shift in the acyl chain composition of both ceramides and sphingomyelins toward shorter acyl chains (C16:0, C18:0, and C18:1) in the ACC was observed. Changes in fatty acyl chain lengths of ceramides have been shown to affect apoptotic pathways, mitochondrial function, and membrane order (Ben-David and Futerman, 2010; Grosch et al., 2012), which are all factors that play a role in PD pathology.

In a broader and somewhat contradicting lipidomic analysis of the occipital cortex in sporadic PD cases, significant changes in 79 sphingolipid, glycerophospholipid, and cholesterol species were detected compared to controls (Cheng et al., 2011). Six out of seven oxysterols analyzed were also increased in the PD visual cortex. This is an interesting finding, as it indicates that changes in lipid metabolism are occurring in the occipital cortex, although this is a brain region which is spared in PD, including the absence of Lewy bodies. The occipital cortex may therefore represent a novel therapeutic target for treating symptoms such as visual hallucinations in PD patients.

Changes in more complex sphingolipids, specifically gangliosides, have also been observed in PD. One study showed a trend of higher GM2 and GM3 in the putamen of PD brains compared to controls (Gegg et al., 2015). Of note, GM1 showed the opposite trend, with significantly lower levels observed in PD patients compared to non-PD controls (Wu et al., 2012). While lipidomic mass spec analysis does not exist for GM1 levels, immunohistochemical staining specific for GM1 of the substantia nigra showed lower levels in both dopaminergic and non-dopaminergic neurons in PD patients versus non-PD controls (Wu et al., 2012). GM1 plays a direct role in regulating calcium homeostasis (Shield et al., 2006) as well as in promoting the integrity of lysosomes (Wei et al., 2009). Furthermore, as discussed already, GM1 prevents the aggregation of α-syn by maintaining its helical conformation (Martinez et al., 2007).

### Lipidomic Analysis of Pathological Lipid Changes in the Plasma Lipidome of PD Patients

All of the lipid alterations mentioned in this section have been observed in different postmortem brain tissues. For these changes to be ultimately useful as biomarkers, the peripheral lipidome should reflect these variations in PD patients. Chan et al. reported elevated levels of GM3 in plasma of PD patients (Chan et al., 2017), which is in line with the observed trends in PD brain compared to controls seen by Gegg et al. (2015), which was mentioned earlier. Mielke et al. (2013) measured levels of ceramide, monohexosylceramides, and lactosylceramides in cognitively normal PD patients, cognitively impaired PD patients, and controls and found that levels of all the lipids in the subclasses described were increased in PD patients, with most ceramide and monohexosylceramide species being higher in those with cognitive impairment compared to those without. As will be discussed in more detail in the following section, mutations in the *GBA* gene which encodes for glucocerebrosidase are strongly associated with PD. A large recent study analyzing the serum lipid levels in 415 PD patients with or without mutations in *GBA* reported that monohexosylceramides, ceramides, and sphingomyelins were higher in patients with *GBA* mutations (Guedes et al., 2017). Of note, this study did not include healthy controls in their analysis. These findings indicate that these lipids play an important role in the pathogenesis of PD, as they were reported to be higher also in patients without *GBA* mutations. Furthermore, PD patients with these mutations are more likely to develop PD earlier in life and to experience dementia and cognitive impairment (Neumann et al., 2009; Brockmann et al., 2011), which could potentially be explained by the accumulation of even higher levels of these lipids in these patients. The next section will discuss mutations in this gene in detail.

### Lipidomic Analysis of Potentially Protective Lipids

Lipidomic analysis tools are not only of use in identifying pathological markers, but also for determining potentially protective lipids. A number of case-control studies have suggested that higher levels of serum cholesterol could be related to a lower prevalence of PD (Scigliano et al., 2006; Miyake et al., 2010). This hypothesis was further supported by three independent prospective studies (de Lau et al., 2006; Simon et al., 2007; Huang et al., 2008), although de Lau and colleagues reported that this association only held true for women but not for men. A recent study following 261,638 statin-free individuals over time also showed that higher levels of both total and low-density lipoprotein cholesterol corresponded with a decreased risk of PD among men (Rozani et al., 2018). Another study which analyzed the data obtained from the Deprenyl and Tocopherol Antioxidative Therapy of Parkinsonism (DATATOP) trial reported that higher serum cholesterol levels were associated with a slower rate of PD clinical progression (Huang et al., 2011). These findings are in line with an early study by Musanti et al. (1993) showing that fibroblasts from PD patients could only incorporate approximately one quarter of the levels of 14C-acetate into cholesterol as control fibroblasts, with cholesterol esterifying activity being reduced by half (Musanti et al., 1993). However, a study based on a Finnish National Insurance Register contradicted the above findings and reported that high total cholesterol is associated with an increased risk of PD in the future (Hu et al., 2008).

Systems based approaches such as lipidomics can be very useful for generating novel hypotheses with regards to pathomechanistic changes in complex diseases. However, one of the challenges that arise with such encompassing data is determining which changes are relevant to the pathology of the disease and not simply natural variations between patients. What the data concerning lipid pathways in PD allows us to conclude thus far is that the changes are lipid species specific, and not just lipid class-related, indicating the importance of analyzing individual lipid species and not just the lipid classes as a whole. Interestingly, lipid changes in the peripheral lipidome of PD patients also generally correspond with changes seen in the brain. This is a promising early step for the development of sensitive and specific biomarkers which can quickly allow for the diagnosis or therapeutic monitoring of PD.

## Mutations in GBA Promote Toxic Conversion of α-Synuclein

Mutations in the *GBA*gene, which encodes the lysosomal enzyme GCase, have been shown to be strongly associated with PD and are the most common risk factor for PD and DLB, found in 25% of all DLB and greater than 10% of PD patients (Clark et al., 2010; Tsuang et al., 2012) GCase produces ceramide and sphingosine by hydrolyzing glucosylceramide (GlcCer) and glucosylsphingosine (GlcSph), respectively. GCase activity is moderately reduced to 58% of normal levels in *GBA* carriers with PD and 67% of normal levels in sporadic PD patients (wild-type *GBA*) suggesting a common pathology accelerated by genetic predisposition (Gegg et al., 2012). However, reduction of GCase activity alone is not sufficient to enhance PD risk, further indicating that precise lipid composition is critical to promote cognitive decline. *GBA* mutations, for example, are also the genetic determinant of Gaucher Disease. In Gaucher disease, homozygous *GBA* mutations reduce GCase activity to <15% of normal function. Yet, only a small subset of Gaucher patients develop PD or DLB (Sidransky, 2012). Moreover, it is only the moderate reductions (as are seen in PD and DLB,) and not the severe reductions in GCase activity (as are observed Gaucher disease), that increase α-syn levels *in vitro* as detected in post-mortem PD brain tissue (Murphy et al., 2014; Kim et al., 2018). The loss of GCase activity has also been measured in the cerebrospinal fluid and blood of PD patients (Alcalay et al., 2015; Parnetti et al., 2017).

The exact pathomechanism by which GCase dysfunction increases the risk of PD still remains elusive, although there have been a number of mechanisms proposed. One proposed hypothesis is a gain-of-function mechanism by which dysfunctional GCase directly interacts with α-syn, promoting its aggregation and accumulation (Sidransky and Lopez, 2012). This hypothesis is supported by the finding that most mutant *GBA* alleles result in a misfolded protein whose new confirmation could promote the aggregation of α-syn. The misfolded protein could also impair autophagy and cause lysosomal dysfunction (Sidransky and Lopez, 2012). Using an ELISA assay, Cullen et al. showed that an increase in α-syn levels was contingent on the levels of mutant *GBA*, but not on the activity (Cullen et al., 2011). Semi-quantitative Western blotting showed a reciprocal relationship between decreased GCase and increased α-syn levels (Gegg et al., 2012; Murphy and Halliday, 2014). However, some *GBA* mutations which have been identified in patients with PD are null mutations, which conflicts with this hypothesis. Additionally, carriers of the null allele may actually have a higher risk of developing PD (Gan-Or et al., 2009). Furthermore, as indicated above, patients with Gaucher disease with homozygous mutations in *GBA1* exhibit an even lower level of GCase activity, although most of them never develop PD (Rosenbloom et al., 2011).

An alternative proposed mechanism is that loss of GCase function leads to accumulation of its substrates, thereby affecting α-syn aggregation and clearance (Mazzulli et al., 2011; Westbroek et al., 2011; Sidransky and Lopez, 2012; Taguchi et al., 2017). Suzuki et al. (2015) knocked down the *Drosophila* homolog of *GBA1* (*dGBA1*) and found that this accelerated the accumulation of proteinase K (PK)-resistant α-syn, which correlated with phenotype severity. GlcCer, a substrate of GCase composed of multiple molecular species, was furthermore shown to directly promote the production of PK-resistant α-syn (Suzuki et al., 2015). Gundner et al. (2018) showed that increased GlcSph levels, another substrate of GCase, mediated an increase in the ratio between α-syn phosphorylated at Serine-129 and total α-syn in the substantia nigra. The phosphorylation of α-syn at residue Ser129 has been correlated with the severity of PD pathology (Lue et al., 2012; Walker et al., 2013), with more than 90% of α-syn in Lewy bodies being phosphorylated at this residue (Fujiwara et al., 2002; Anderson et al., 2006). Using path analysis to define six hypothetical models for describing the impact of GCase and α-syn on PD status, the authors showed that GlcSph is not the only mediator according to the model, with changes in glucocerebrosidase also promoting α-syn accumulation via alternative mechanisms (Gundner et al., 2018). Using a Gaucher’s disease mouse model with a knock-in point mutation (D409V/D409V), Sardi et al. (2011) reported that α-syn accumulation was more closely correlated with the levels of glucosylsphingosine than glucosylceramide, and that these mice developed a corresponding memory deficit. The authors could reverse this deficit by administering recombinant GCase directly into the brain. However, neuropathological changes were observed in both the D409V homozygote and heterozygote mice. This suggests that GCase activity and consequent substrate accumulation do not fully explain α-syn aggregation. In a Gaucher mouse model homozygous for V394L or D409H, crossed with mice deficient in the peptide saposin C, a co-factor necessary for GCase activation, α-syn aggregated in cortical neurons, but this did not correlate with glucosylceramide accumulation (Xu et al., 2011). Lastly, Gegg et al. (2012) reported that there was no accumulation of either GlcSph or GlcCer in the putamen or cerebellum of patients with heterozygous mutations in *GBA*. These findings all weaken the loss-of-function hypothesis.

The third hypothesis represents a bi-directional feedback loop between GCase and α-syn, where oligomeric α-syn interferes with GCase trafficking and activity, which further exacerbates α-syn pathology (Mazzulli et al., 2011). Mazzulli et al. (2011) demonstrated that functional loss of GCase activity by GCase shRNA-mediated knock-down causes the accumulation of α-syn and results in aggregation-dependent neurotoxicity in dopaminergic neurons derived from induced pluripotent stem cells (iPSCs). The α-syn aggregation was not accompanied by a change in mRNA levels, suggesting that the increased protein levels were due to impaired degradation. They furthermore showed that the lysosomal activity of GCase in neurons and the brains of patients with sporadic PD was inhibited by α-syn, indicating the presence of a positive feedback loop that could perpetually propagate the disease. Further supporting this hypothesis, it was recently demonstrated that the abundance of aggregates of a newly identified high molecular weight α-syn (24 kDa) linearly correlated with the loss of GCase function (Brekk et al., 2018).

These three hypotheses, however, do not consider the crosstalk between GCase and other enzymes involved in lipid metabolism. Recent reports suggest that there is constructive interference between GCase and β-Gal activity contributing to α-syn aggregation and toxicity in PD. Schondorf et al. (2014) reported that neurons derived from PD patient iPSCs had both decreased GCase activity and a reduced β-Gal activity, which were both rescued using a zinc-finger nuclease-mediated gene correction. Further supporting this novel hypothesis, patients with β-galactosialidosis, another lysosomal storage disorder, who have a deficiency in β-Gal activity have been reported to have α-syn accumulation in the brain (Suzuki et al., 2007; Hamano et al., 2008).

These data lead us to hypothesize that an individual’s lipid metabolic response to GBA mutations, possibly influenced by diet, drives cognitive decline and disease risk. This alternate “metabolic” hypothesis is further strengthened by evidence that the p.L302P loss of function mutation in *SMPD1* increases risk of PD in persons of Ashkenazi Jewish and Chinese Han ancestry (Gan-Or et al., 2013; Dagan et al., 2015; Mao et al., 2017). *SMPD1* encodes for lysosomal acid sphingomyelinase (SMase) which hydrolyzes sphingomyelins to ceramides and phosphocholine. Finally, *GALC* encodes galactosylceramidase (GalC) responsible for the hydrolysis of galactosylceramides (GalCers) to galactose and ceramides (Kobayashi et al., 1986). Again, loss of enzymatic function increases α-syn aggregation and accumulation in human cells and experimental murine models (Xu et al., 2011; Bae et al., 2015). This indicates that underlying metabolic differences in ceramide homeostasis modulate susceptibility and resilience to the loss of function of these and other lysosomal enzymes. The critical lipid players in this hypothesis are the ceramides. Intriguingly, the Krainc laboratory has elegantly demonstrated that the α-syn accumulation associated with *GBA* loss of function can be prevented *in vitro* by restoring Cer(d18:1/18:0)^2^ levels to baseline (Kim et al., 2018). Cer(d18:1/18:0) levels fall following GCase inhibition *in vitro* (Kim et al., 2018). This decline is further compounded when Cer(d18:1/18:0)is hydrolyzed to sphingosine and stearic acid (18:0) by lysosomal acid ceramidase (*ASAH1)*. Inhibition of the lysosomal acid ceramidase activity, in the presence of GCase impairment, rescues ceramide levels and prevents α-syn accumulation *in vitro* (Kim et al., 2018). These data help to reconcile genetic evidence that not all carriers of risk-associated *GBA* or *SMPD1* mutations develop PD, PDD, or DLB (Schlossmacher et al., 2017). Building on this hypothesis, we suggest that an individual’s sphingolipid metabolome confers susceptibility (or resistance) to PDD and DLB phenoconversion via molecular modulation of ceramide homeostasis. Clearly, testing this hypothesis will require mapping of disease-specific metabolic impairments and interventions designed to restore metabolic homeostasis.

## Other PD Genetic Risk Factors Associated With Lipid Metabolism and Trafficking

In addition to the genes reviewed above, a number of loci in other genes associated with normal lipid function, metabolism, and trafficking (Manzoni and Lewis, 2013; Clague and Rochin, 2016) have been identified to increase the susceptibility of developing PD (Klein and Westenberger, 2012; Nalls et al., 2014).

### PLA2G6

Mutations in pleiotropic lipid regulators involved in sphingolipid and glycerophospholipid metabolism, *PLA2G6*, have been found to cause levodopa-responsive parkinsonism with dystonia (Doherty and Hardy, 2013). *PLA2G6*-associated PD is caused by the homozygous or compound heterozygous inheritance of various missense mutations in this gene (Paisan-Ruiz et al., 2009; Sina et al., 2009; Yoshino et al., 2010). The *PLA2G6* gene encodes for calcium-independent phospholipase (iPLA2) which catalyzes the hydrolysis of fatty acids from glycerophospholipids (Baburina and Jackowski, 1999; Barbour et al., 1999). *PLA2G6* knockout in *Drosophila* resulted in mitochondrial dysfunction, increased lipid peroxidation, and neurodegeneration, and fibroblasts from a patient with the PD-associated p.R747W mutation showed similar mitochondrial impairment (Kinghorn et al., 2015). Furthermore, it has been shown that activation of *PLA2G6* promotes the hydrolysis of sphingomyelins by neutral sphingomyelinase, thereby resulting in increased levels of ceramides (Lei et al., 2007). Therefore, mutations in *PLA2G6* reducing its activity would lead to lower ceramide levels, once again linking an impaired ceramide metabolism with the development of PD.

### SCARB2

The *SCARB2* locus was also found to be associated with PD by Simon-Sanchez et al. (2009), which was later confirmed by two other genome wide association studies (GWAS) (Do et al., 2011; Nalls et al., 2014). This gene encodes a GCase receptor called lysosomal membrane protein 2 (LIMP-2) which is responsible for directing GCase to lysosomes (Redensek et al., 2017). A deficiency in LIMP-2 can cause a decrease in GCase activity and in the degradation of α-syn (Gan-Or et al., 2015), and could therefore potentially lead to a decrease in ceramide levels, lending yet more support to this hypothesis.

### SREBF1

The SREBF1 locus was first found to be associated with PD susceptibility in 2011 through a genome wide association study (Do et al., 2011), which was then confirmed by Nalls et al. (2014) by the meta-analysis of GWASs. Using a genome-wide RNAi screen to identify genes involved in the regulation of the PINK1/Parkin pathway, Ivatt et al. (2014) further showed that SREBF1 is involved in regulating the autophagic degradation of mitochondria, known as mitophagy.

*SREBF1* encodes sterol regulatory element-binding protein 1 (SREBP-1), which is a transcriptional factor involved in lysosomal lipid accumulation. Gan-Or et al. (2015) reported that reduced *SREBF1* expression downregulates the *NPC1* genes and leads to the accumulation of cholesterol in lysosomes and late endosomes. As mentioned, *SREBF1* also plays a role in mitophagy. Knockdown of *SREBF1* was found to decrease mitophagy by blocking the translocation of Parkin into the mitochondria (Gan-Or et al., 2015), and this effect could be rescued by the addition of exogenous cholesterol and fatty acids. Ivatt and Whitworth (2014) added on to these results by looking upstream of Parkin translocation. *PINK1* becomes stabilized on the outer mitochondrial membrane upon injury to the mitochondria, and chemical inhibition or silencing of *SREBF1* resulted in decreased *PINK1* stabilization (Ivatt and Whitworth, 2014). These findings suggest that genetic variation at the SREBF1 locus may affect its expression and could consequently lead to reduced *PINK1* stabilization on the outer mitochondrial membrane and disrupted mitochondrial homeostasis. Addition of exogenous lipids showed a trend toward rescuing *PINK1* stabilization (Ivatt and Whitworth, 2014). Although none of the differences seen were statistically significant, these results indicate that lipid synthesis could potentially play an important role in mitochondrial homeostasis, and dysregulation of lipid metabolic pathways could promote PD pathogenesis.

### DGKQ

Diacylglycerol kinase theta (*DGKQ*) has been suggested as a susceptibility gene for PD in an early GWAS study (Pankratz et al., 2009), with two additional GWASs providing further support for its involvement in PD (Simon-Sanchez et al., 2009; UK Parkinson’s Disease Consortium et al., 2011). Diacylglycerol kinases are responsible for phosphorylating phosphatidic acid, thereby producing DAG. As described in an earlier section, DAG levels were found to be elevated in PD patients without specifically reported mutations, indicating that dysfunction in DAG kinase is involved in PD pathology irrespective of the presence of a mutation. This is in line with a number of reports that DAG is implicated in directly promoting the oligomerization of α-syn as well as being involved in glycerophosphoinositol turnover and lipid signaling. Furthermore, diacylglycerol kinases have been demonstrated to affect the trafficking and fusion of vesicles at synaptic terminals (Merida et al., 2008).

These reports suggest that it may be worthwhile to consider other genes pertaining to enzymes involved in lipid metabolism as not only risk factors for PD, but also as clues to deciphering the full involvement of lipids in the pathogenesis of this disease.

## Lipid Dysregulation Leading to Oxidative Stress and Inflammation in Parkinson’s Disease

There is increasing evidence that oxidative stress and inflammatory processes play a role in the pathogenesis of PD (Beal, 2003; Jenner, 2003; McGeer and McGeer, 2004; Abou-Sleiman et al., 2006; Schapira, 2008; Hirsch and Hunot, 2009; Hirsch et al., 2012; Joshi and Singh, 2018). Oxidative stress is characterized by an increase in reactive oxygen species (ROS) which overpower antioxidant mechanisms and result in cytotoxicity (Halliwell, 2006). The mitochondrial respiratory chain, uncontrolled ARA cascade, and NADPH oxidase are the major sources of ROS, utilizing molecular oxygen to produce ROS. Hydroxyl radicals are also produced during these processes through the Fenton reaction. These radicals can then form peroxyl radicals (ROO^∙^) by attacking polyunsaturated fatty acids in membrane glycerophospholipids, propagating the lipid peroxidation chain reaction (Farooqui, 2010b).

Markers of oxidative damage such isoprostanes, hydroxyeicosatetraenoic acid products (HETEs), and cholesterol oxidation products were found to be increased in PD patients compared to controls. The same authors also reported that the enzymatic activities of platelet activating factor-acetylhydrolase (PAH-AH) were significantly lower in PD patients (Seet et al., 2010). This is of importance, as PAF-AH is inhibited by oxygen radicals (Ambrosio et al., 1994). A number of other lipid peroxidation markers, including isofurans, 4-hydroxy-trans-2-nonenal (4-HNE), and 4-oxo-trans-2-nonenal (4-ONE) have also been reported to be significantly increased in PD patients compared to controls (Farooqui and Farooqui, 2011). These metabolites are derived from ARA, which as mentioned earlier is released from glycerophospholipids by cytosolic PLA2. Interestingly, mice deficient in cPLA2 were found to be resistant to MPTP-induced dopaminergic neurotoxicity (Klivenyi et al., 1998).

When PLA2 releases ARA from glycerophospholipids, the other product of this reaction are lysophospholipids. These can be remodeled via the Lands cycle to PAFs, pro-inflammatory factors that act to increase the intensity of the inflammatory response (Farooqui, 2010a). Most PAFs act through a G-protein coupled receptor called platelet activating factor receptor (PAF-R) (Bito et al., 1994). A study by Kim and colleagues showed that treating mice with MPTP significantly increased the levels of PAFs in the striatum, specifically the PC(O-18:1:2:0) PAF, as well as increased the expression of PAF-R (Kim et al., 2013). In mice treated with ginkgolide B, an inhibitor of PAF-R, or in PAF-R knock-out mice, MPTP-induced dopaminergic neurodegeneration was attenuated. These studies indicate that the effects of oxidative stress and the inflammatory response involve an interplay between numerous lipid mediators, the enzymes involved in lipid processing, as well as associated receptors of these molecules.

## Lipids as Either Treatment or Treatment Targets in Parkinson’s Disease

As discussed, altered lipid pathways and lipid mebrane composition in PD appear to play significant roles in disease pathogenesis and thus present promising targets for PD treatment. Here we will briefly discuss studies describing the use of lipids or of lipid analogs as treatments for PD, as well as modulators of enzymes involved in lipid metabolism.

### Gangliosides and Ganglioside Analogs

In MPTP mouse models of PD, treatment with the ganglioside GM1 was shown to partially restore depleted levels of dopamine and promote neuron recovery (Hadjiconstantinou et al., 1986, 1989; Schneider et al., 1995). Furthermore, in MPTP primate models of PD, GM1 treatments restored dopaminergic terminals in the striatum (Pope-Coleman et al., 2000) and led to significant recovery of motor functions (Pope-Coleman and Schneider, 1998). A randomized delayed start trial including 77 patients with PD reported that treatment with GM1 for 120 weeks compared to a 24-week delayed start and subsequent treatment for 96 weeks showed significant imporvement in motor scores as well as sustained benefit after the end of the trial (Schneider et al., 2013). A subsequent 5-year open study in which PD patients received GM1 found that there was an improvement in motor symptoms compared to baseline, although this was somewhat modest (Schneider et al., 2010). This suggests that GM1 has beneficial effects with regards to PD symptoms and potentially disease progression, which is in line with the previously mentioned studies showing that PD patients have a GM1 deficiciency (Wu et al., 2012), and that GM1 inhibits the formation of α-syn fibrils (Martinez et al., 2007).

The beneficial effects of GM1 treatment in these studies may have been compromised by the limited access GM1 had to the brain and furthermore to the neurons themselves. Much greater improvements may be possible through the use of membrane-permeable GM1 analogs that could easily cross the blood-brain barrier. Such an analog of GM1, LIGA-20, was developed in 1990 by Manev et al. (1990). It has the same ologosaccharide chain as GM1, but contains a modified hydrophobic moiety which allows it to be permeable to the plasma membrane. This analog proved to be effective via oral administration and appeared to be much more potent than GM1 in promoting the recovery of dopamine levels in the striatum in an MPTP mouse model of PD (Schneider and DiStefano, 1994; 1995).

### Statins

There have been a number of reports suggesting that the use of statins, cholesterol-lowering drugs, may prevent the development of PD. A study by Wahner and colleagues reported a high frequency of statin use in the 342 healthy controls recruited versus 312 sporadic PD patients (Wahner et al., 2008). Furthermore, a recent meta-analysis investigated the use of statins with regard to the risk of developing PD and concluded that statin use was associated with a lower risk of PD (Bai et al., 2016). As statins are known to lower cholesterol levels, this somewhat contradicts the reports from several case-control studies mentioned earlier which identified higher levels of cholesterol as potentially protective (Scigliano et al., 2006; Miyake et al., 2010). One explanation for this could be that the beneficial anti-inflammatory or anti-oxidant effects of statins could compensate for the decreased cholesterol levels.

### Phospholipase A2 Inhibitors

The development of novel inhibitors of phospholipase A2 (PLA2) have also proven promising in treating PD. Yoshinaga et al. (2000) showed that an inhibitor of cPLA2 (arachidonyl trifluoromethyl ketone) reduced MPTP-induced cytotoxicity in a GH3 cell line, a model for dopaminergic neurons derived from rat anterior pituitary. Inhibition of PLA2 by quinicrine *in vivo* in mice was also demonstrated to have protective effects on MPTP-induced depletion of striatal dopamine in a dose-dependent manner (Tariq et al., 2001).

Further research into lipid alterations in PD could give rise to a wide array of novel treatments based on the specific lipid species, enzymes, or overall lipid pathways and networks found to be causative factors in PD. Clearly, a relevant focus is substrate reduction and enzymatic replacement therapies focused on regulating ceramide and GlcCer homeostasis.

## Conclusion

To be able to prevent PD or at the least to combat it more effectively in the future, the interplay between multiple pathomechanisms involved must be elucidated. Current PD treatments only manage symptoms and are unable to impede disease progression. PD patients do not experience motor symptoms until more than 50% of the neurons in the substantia nigra have degenerated (Bernheimer et al., 1973), which results in a substantial lag in treatment. Therefore, it is imperative to identify molecular changes that can be measured very early in the course of the disease. Changes in membrane lipids have been observed in both affected and unaffected regions of brains from PD patients, indicating that these lipid pathway alterations may precede Lewy body pathology. Identifying these early changes in lipids could help in diagnosing the disease earlier and employing neuroprotective therapies, discrimating patients with PD from those with other similar neurodegenerative disorders such as DLB, as well as stratifying PD patients and providing them with personalized treatment. Different steps in lipid metabolism could be targeted to modulate levels of both toxic lipids and potentially protective lipids which in the future could lead to more effective treatments with fewer side effects.

## Author Contributions

IA and SB performed the literature review and wrote the manuscript.

## Conflict of Interest Statement

The authors declare that the research was conducted in the absence of any commercial or financial relationships that could be construed as a potential conflict of interest.

## Footnotes

1 Lipid nomenclature describes the molecular identity of phospholipids as follows: PC refer to a lipid with a glycerophosphocholine head group. O- indicates an ether linkage; P- indicates a vinyl ether linkage; no designation refers to an ester linkage to the glycerol backbone. PC(O-16:0/2:0) refers to a molecular species with a hydrocarbon chain of 16 carbons and no unsaturations linked to the phosphoglycerate backbone by an ether/alkyl linkage at the *sn-*1 position and a 2 carbon acetyl chain at the *sn-*2 position with no unsaturations.

2 Lipid nomenclature describes the molecular identity of each sphingolipid as follows: Cer refers to a lipid within the ceramide family. d18:1 refers to a ceramide with a sphingosine backbone. 18:0 refers to a particular molecular species with an *N*-acyl hydrocarbon chain of 18 carbons and no unsaturations (:0).
